# Rapid Increase of Community SARS-CoV-2 Seroprevalence during Second Wave of COVID-19, Yaoundé, Cameroon

**DOI:** 10.3201/eid2806.212580

**Published:** 2022-06

**Authors:** Francis Ateba Ndongo, Emilande Guichet, Eric Donald Mimbé, Justin Ndié, Raphael Pelloquin, Marie Varloteaux, Livo Esemu, Mireille Mpoudi-Etame, Nadine Lamare, Ginette Edoul, Rodrigue Kamga Wouambo, Dowbiss Meta Djomsi, Marcel Tongo, Félicité Naah Tabala, Rogacien Kana Dongmo, Mamadou Saliou Kalifa Diallo, Julie Bouillin, Guillaume Thaurignac, Ahidjo Ayouba, Martine Peeters, Eric Delaporte, Anne-Cécile Zoung-Kanyi Bissek, Eitel Mpoudi-Ngolé

**Affiliations:** Ministry of Public Health of Cameroon Division of Operational Research in Health, Yaoundé, Cameroon (F. Ateba Ndongo, J. Ndié, F.N. Tabala, R.K. Dongmo, A.-C. Zoung-Kanyi Bissek);; Recherches Translationnelles sur le VIH et les Maladies Infectieuses, University of Montpellier, Institut de Recherche pour le Développement, Institut National de la Santé et de la Recherche Médicale, Montpellier, France (E. Guichet, R. Pelloquin, M.S.K. Diallo, J. Bouillin, G. Thaurignac, A. Ayouba, M. Peeters, E. Delaporte);; French National Agency for Research on AIDS and Infectious Diseases, Cameroon Site, Central Hospital of Yaoundé, Yaoundé (E.D. Mimbé, M. Varloteaux, A.-C. Zoung-Kanyi Bissek, E. Mpoudi-Ngolé);; Centre de Recherche sur les Maladies Emergentes et Réémergentes, Yaoundé (L. Esemu, N. Lamare, G. Edoul, R. Kamga Wouambo, D. Meta Djomsi, M. Tongo, E. Mpoudi-Ngolé);; Military Hospital of Yaoundé, Yaoundé (M. Mpoudi-Etame)

**Keywords:** COVID-19, SARS-CoV-2, antibodies, seroepidemiologic studies, Cameroon, coronavirus disease, severe acute respiratory syndrome coronavirus 2, viruses, respiratory infections, zoonoses

## Abstract

We conducted 2 independent population-based SARS-CoV-2 serosurveys in Yaoundé, Cameroon, during January 27–February 6 and April 24–May 19, 2021. Overall age-standardized SARS-CoV-2 IgG seroprevalence increased from 18.6% in the first survey to 51.3% in the second (p<0.001). This finding illustrates high community transmission during the second wave of COVID-19.

Since the recognition of the first cases of COVID-19 at the end of 2019 in Wuhan, China, SARS-CoV-2 has spread rapidly across the globe. By late November 2021, almost 260 million confirmed cases, including at least 5 million deaths, had been reported (*1*). Cases from Africa represent only 3.4% of those cases worldwide (*1*,*2*), but serologic surveys demonstrate that the extent of SARS-CoV-2 spread in Africa is higher (*3*). After the first pandemic wave, overall seroprevalence in Africa was estimated at ≈22%, ranging from <1% to >70% depending on country and study population (*3*). The few studies reporting data after the second wave in Africa demonstrated a rapid increase to >50% seroprevalence (*4*–*6*). Underestimation of COVID-19 cases was most likely caused by weak healthcare infrastructure, low or no access to diagnostic testing, and higher proportions of paucisymptomatic or asymptomatic disease related to younger population or cross-reactive immunity from other coronavirus infections. The overall objective of our study was to evaluate the effect of the second wave of COVID-19 on SARS-COV-2 seroprevalence in the general population of Yaoundé, the capital city of Cameroon.

## The Study

We conducted 2 population-based seroprevalence surveys in Yaoundé during January 27–February 6, 2021 (survey 1) and April 24–May 19, 2021 (survey 2). We adapted the study design from the World Health Organization population-based age-stratified seroepidemiologic investigation protocol for COVID-19 infection, version 2.0 (*7*). We randomly selected households in 6 of the 7 health districts in Yaoundé, with a probability of being selected proportional to the population number in each enumeration area (Appendix Figure 1). In 50% of households, we invited all residents to participate; among the remaining 50%, we invited only residents >40 years of age. We calculated sample size to estimate overall seroprevalence in Yaoundé. The samples were independent for the 2 surveys. All persons belonging to the selected household were eligible. We scheduled appointments for participants who were absent during the survey. We used individual questionnaires to collect sociodemographic data, medical history associated with COVID-19 symptoms (in the 4 months before the start of the survey), contact with COVID-19 patients, and previous SARS-CoV-2 tests (recall period beginning in March 2020). We offered PCR testing to all participants who were suspected to be SARS-CoV-2–positive. We obtained written consent from all adults and written parental consent for participants <21 years of age (with children’s assent when >10 years of age). The study was approved by the national ethics committee (approval no. 2020/10/1310/CE/CNERSH/SP).

We collected whole blood samples in EDTA tubes and as dried blood spot (DBS) samples for children and other participants who declined to provide venous blood. We eluted DBS samples and used 100 μL of diluted eluate, adjusted at a final plasma dilution of 1/200, as previously validated (Appendix Figure 2), to test for SARS-CoV-2 antibodies with a previously developed, highly sensitive, and specific multiplex assay (Luminex Corporation, https://www.luminexcorp.com) using recombinant nucleocapsid (NC) and spike (SP) SARS- CoV-2 proteins (*8*). We considered samples positive when they reacted simultaneously with NC and S proteins but considered samples reacting with only 1 antigen indeterminate because of the difficulty discriminating between antibody decline or lower specificity of single-antigen reaction, especially with samples from populations in Africa (*9*,*10*). The test was previously evaluated on 1,197 samples from Africa before the COVID-19 pandemic, including 184 from Cameroon, with 99.7% specificity (*11*).

We performed statistical analysis with Stata 16 (StataCorp, https://www.stata.com). We age-standardized the overall seroprevalence estimate on the basis of available demographic data (*12*) and tested associations between positive serologic tests and key risk factors with multivariate logistic models and likelihood ratio tests. We used the Pearson χ^2^ test to compare categorical descriptive outcomes.

In the first survey, 786 (47.7%) of 1,647 eligible participants from 392 households were included. For 722 persons, we obtained sufficient sample volume for antibody testing. To improve participation for the second survey, we strengthened community mobilization, conducted surveys on the weekend, and scheduled appointments for absent participants. In the second survey, 1,234 (85.3%) of 1,447 eligible persons from 424 households were included. Serologic data were available for 1,228 persons. Distribution of sex was comparable between the surveys; the proportion of participants <20 years of age was higher but not significantly so in the second survey (Table 1). Approximately 15% of participants reported a previous diagnostic SARS-CoV-2 PCR test; only 1.3% (1/77) reported a positive test in the first survey and 2.1% (4/194) in the second survey. In both surveys, a limited number of participants (3.3% in the first survey, 4.1% in the second) reported contact with a PCR-confirmed SARS-CoV-2–positive person.

**Table 1:** Sociodemographic characteristics of participants in study of community SARS-CoV-2 seroprevalence during second wave of COVID-19 epidemic, by sex, Yaoundé, Cameroon, 2021*

Characteristic	Survey 1, January 27–February 6				Survey 2, April 24–May 19		
	Female	Male	Total		Female	Male	Total
Age group, y	n = 786				n = 1,234		
0–19	132 (28.3)	123 (37.7)	255 (32.2)		261 (36.3)	208 (40.8)	469 (38.0)
20–39	205 (44.7)	103 (31.8)	308 (39.3)		278 (38.6)	165 (32.1)	443 (35.9)
>40	124 (27.0)	99 (30.7)	223 (28.5)		181 (25.1)	141 (27.4)	322 (26.1)
Marital status	n = 638				n = 1,216		
Single	186 (48.1)	133 (54.0)	319 (50.0)		442 (62.4)	365 (71.9)	807 (66.4)
Married or living as a couple	158 (40.8)	109 (43.4)	267 (41.9)		216 (30.5)	132 (26.0)	348 (28.6)
Divorced or separated	37 (9.6)	3 (1.2)	40 (6.3)		43 (6.1)	10 (2.0)	53 (4.4)
Widower or widow	6 (1.6)	6 (2.4)	12 (1.9)		7 (1.0)	1 (0.0)	8 (1.0)
Education	n = 681				n = 1,227		
None	26 (6.4)	11 (4.0)	37 (5.4)		75 (10.5)	45 (8.8)	120 (9.8)
Primary school	81 (19.8)	52 (19.1)	133 (19.5)		197 (27.6)	137 (26.8)	334 (27.2)
Secondary school	213 (52.1)	131 (48.2)	344 (50.5)		323 (45.2)	203 (39.7)	526 (42.9)
University	89 (21.8)	78 (28.7)	167 (24.5)		120 (16.8)	127 (24.8)	247 (20.1)
Profession	n = 620				n = 1,192		
Student	100 (26.6)	75 (30.7)	175 (28.2)		242 (34.9)	201 (40.4)	443 (37.2)
Sales or service	67 (17.8)	46 (18.9)	113 (18.2)		145 (20.9)	70 (14.1)	215 (18.0)
Women or men at home	102 (27.1)	0 (0.0)	102 (16.5)		126 (18.2)	4 (0.8)	130 (10.9)
Professional or manager	40 (10.6)	28 (11.5)	68 (11.0)		59 (8.5)	52 (10.4)	111 (9.3)
Construction	0 (0.0)	16 (6.6)	16 (2.6)		1 (0.0)	9 (1.8)	10 (0.8)
Unemployed	20 (5.3)	21 (8.6)	41 (6.6)		71 (10.2)	65 (13.1)	136 (11.4)
Other	47 (12.5)	58 (23.8)	105 (17.0)		50 (7.2)	97 (19.5)	147 (12.3)
Total	461 (58.7)	325 (41.3)	786 (100.0)		720 (58.3)	514 (41.7)	1,234 (100.0)

*Values are no. (%) participants.

The overall age-standardized SARS-CoV-2 IgG seroprevalence against SP and NC proteins increased from 18.6% (95% CI 15.7%–21.7%) to 51.3% (95% CI 48.3%–54.2%) (p<0.001) during the 3-month period between surveys (Table 2). In both surveys, seroprevalence remained comparable between men and women (Table 2). Seroprevalence increased in all age categories and was significantly higher among persons >20 years of age in both surveys (p = 0.002 for survey 1 and p<0.001 for survey 2). The proportion of persons with SP protein antibodies only (29.1% vs. 16.9%) was higher than those with NC antibodies only (5.8% vs. 5.2%) (Appendix Table 1). We determined population-level distributions of median fluorescence intensity for each of the SARS-CoV-2 antigens (Appendix Figure 3). We found no association between seropositivity and history of symptoms associated with COVID-19 or hospitalization in general before the survey (Appendix Table 2).

**Table 2:** Seroprevalence of SARS-COV-2 antibodies by age, sex, and medical history in 2 consecutive population-based surveys during second wave of COVID-19, Yaoundé, Cameroon, 2021*

Characteristic	Participants, survey 1, January 27–February 6					Participants, survey 2, April 24–May 19			
	Total no.	No. (%) positive	% Positive (95% CI)	p value		Total no.	No. (%) positive	% Positive (95% CI)	p value
Age group, y				0.002					<0.001
0–19	236	31	13.1 (9.3–18.3)			468	200	42.7 (38.3–47.3)	
20–39	276	71	25.7 (20.8–31.4)			440	263	59.8 (55.0–64.4)	
>40	210	48	22.9 (17.5–29.2)			320	201	62.8 (57.3–68.0)	
Sex				0.773					0.942
F	423	89	18.5 (14.8–22.9)			718	392	51.0 (47.1–54.8)	
M	299	61	19.0 (14.8–24.1)			510	272	51.6 (47.0–56.1)	
No. symptoms				0.688					0.288
0	271	70	22.5 (17.8–28.0)			776	424	51.8 (48.1–55.5)	
1–2	157	26	12.8 (7.5–21.0)			257	129	47.8 (41.6–54.0)	
3–5	134	27	22.5 (13.5–35.2)			167	92	53.9 (45.6–61.9)	
>5	68	17	18.4 (8.4–35.9)			28	19	57.5 (39.0–75.7)	
Hospitalization				0.150					0.487
Yes	28	6	10.7 (4.4–23.6)			12	8	51.0 (17.3–83.9)	
No	329	64	18.0 (12.8–24.7)			445	229	50.1 (45.1–55.2)	
Total	722	150	18.6 (15.7–21.7)			1 228	664	51.3 (48.3–54.2)	

*Overall seroprevalence estimate was age-standardized, based on available demographic data (*12*).

## Conclusions

In these 2 consecutive population-based SARS-CoV-2 seroprevalence studies, conducted just before the start of the second wave of COVID-19 (January–February 2021) and at its decreasing trend (April–May 2021) (Appendix Figure 4), we found extensive community transmission in Yaoundé, where seroprevalence reached up to 50%. By the end of November 2021, Cameroon reported only 106,749 cases (*2*), but seroprevalence suggests that by early May 2021, 51% of the population of Yaoundé had antibodies to SARS-CoV-2, corresponding to >2 million persons in the total population of Yaoundé (estimated to be ≈4.1 million). Choice of serologic tests is vital (*9*), and therefore we used strict criteria and considered seropositivity as presence of antibodies to 2 different SARS-CoV-2 antigens (*8*,*11*). We cannot exclude that a proportion of the participants with antibodies against a single antigen also had a previous SARS-CoV-2 infection or were seroconverting (*10*).

The disparity in numbers of confirmed cases and persons estimated to have SARS-CoV-2 antibodies clearly demonstrates that COVID-19 infections were mainly paucisymptomatic or asymptomatic (*1*,*2*). We also observed no association between history of symptoms or hospitalization. Moreover, few persons reported contact with confirmed SARS-CoV-2–positive persons or had received a PCR test. Similar findings were reported in other studies in Africa (*4*–*6*).

Overall, the results of the household SARS-CoV-2 serosurveys during the second COVID-19 wave in Yaoundé, Cameroon, show a high seroprevalence and rapid spread in the general population similar to that observed in other countries in Africa (*4**–**6*,*13*). The country faced additional waves, and new population-based studies to monitor the evolution of seroprevalences to the different antibodies against SARS-CoV-2 epitopes will be vital. It can also not be excluded that antigens from the different SARS-CoV-2 variants have to be included in future assays, especially against highly divergent variants as illustrated by the emergence of the Omicron variant.

Appendix Additional information about rapid increase of community SARS-CoV-2 seroprevalence during second wave of COVID-19 epidemic, Yaounde, Cameroon
