# Supplementary material for: Rapid Increase of Community SARS-CoV-2 Seroprevalence during Second Wave of COVID-19, Yaoundé, Cameroon
Source: Emerg Infect Dis. 2022 Jun;28(6):1233–6. doi: 10.3201/eid2806.212580 (PMC9155890; doi:10.3201/eid2806.212580)
Supplement: Appendix — Additional information about rapid increase of community SARS-CoV-2 seroprevalence during second wave of COVID-19 epidemic, Yaounde, Cameroon [file 21-2580-Techapp-s1.pdf]

Article DOI: https://doi.org/10.3201/eid2806.212580

Rapid Increase of Community SARS-CoV-2 Seroprevalence during Second Wave of COVID-19 Epidemic, Yaounde, Cameroon

Appendix

Appendix Table 1. Seroprevalence of SARS-COV-2 antibodies, including seropositive and indeterminate status, by age and gender in 2 consecutive population-based surveys at 3-months' interval in Yaounde, Cameroon, during second wave of COVID-19 in 2021*

Characteristic	No. participants	Seropositive (IgG anti-SP+ and anti-NC+)			Indeterminate (IgG anti-SP+ only)			Indeterminate (IgG anti-NC+ only)		
		No. (%)	95% CI	p-value	No. (%)	95% CI	p-value	No. (%)	95% CI	p-value
Survey 1										
Age group, y				0.002			0.565			0.002
0–19	236	31 (13.1)	9.3–18.3		73 (30.9)	25.2–37.3		5 (2.1)	0.9–5.1	
20–39	276	71 (25.7)	20.8–31.4		74 (26.8)	21.8–32.5		27 (9.8)	6.7–14.0	
≥40	210	48 (22.9)	17.5–29.2		58 (27.6)	21.8–34.3		21 (10.0)	6.5–15.0	
Gender				0.773			0.501			0.559
F	423	89 (18.5)	14.8–22.9		122 (30.5)	25.6–35.8		29 (5.1)	3.4–7.5	
M	299	61 (19.0)	14.8–24.1		83 (27.5)	22.3–33.4		24 (6.9)	4.5–10.3	
Total	722	150 (18.6)	15.7–21.7		205 (29.1)	25.5–33.0		53 (5.8)	4.3–7.6	
Survey 2										
Age group, y				<0.001			0.024			0.029
0–19	468	200 (42.7)	38.3–47.3		92 (19.7)	16.2–23.6		16 (3.4)	2.1–5.6	
20–39	440	263 (59.8)	55.0–64.4		64 (14.6)	11.5–18.3		32 (7.3)	5.1–10.2	
≥40	320	201 (62.8)	57.3–68.0		41 (12.8)	9.5–17.0		22 (6.9)	4.5–10.4	
Gender				0.942			0.715			0.158
F	718	392 (51.0)	47.1–54.8		116 (16.7)	14.0–19.9		34 (4.3)	3.0–6.1	
M	510	272 (51.6)	47.0–56.1		81 (17.2)	14.0–21.1		36 (6.4)	4.6–8.9	
Total	1228	664 (51.3)	48.3–54.2		197 (16.9)	14.8–19.4		70 (5.2)	4.1–6.6	

*Overall seroprevalence estimate was age-standardized based on available demographic data (*1*). NC, nucleocapsid; SP, spike protein.

Appendix Table 2. Association between antibodies to SARS-CoV-2 and risk factors: multivariate logistic model, Yaounde, Cameroon, 2021*

	Survey 1 (n = 722)		Survey 2 (n = 1228)			
Characteristic	OR (95% CI)	p-value	OR (95% CI)	p-value	aOR (95% CI)	p-value
Age group, y						
0–19	1 (ref)		1 (ref)		1 (ref)	
20–39	2.29 (1.42–3.69)	0.001	1.99 (1.52–2.61)	<0.0001	1.57 (1.11–2.24)	0.013
≥40	1.96 (1.17–3.27)	0.011	2.26 (1.68–3.04)	<0.0001	1.52 (1.00–2.31)	0.049
Gender						
M	1 (ref)		1 (ref)			
F	1.04 (0.71–1.52)	0.836	1.05 (0.83–1.32)	0.663		
No. symptoms						
0	1 (ref)		1 (ref)			
1 to 2	0.57 (0.34–0.95)	0.031	0.84 (0.63–1.12)	0.222		
3 to 5	0.72 (0.43–1.22)	0.218	1.02 (0.72–1.43)	0.915		
>5	0.96 (0.51–1.78)	0.888	1.75 (0.77–4.01)	0.179		
Hospitalization						
No	1 (ref)		1 (ref)		1 (ref)	
Yes	1.13 (0.43–2.99)	0.802	1.80 (0.52–6.27)	0.347		

*aOR, adjusted odds ratio; OR, odds ratio.

References

1. The World Bank. Cameroon: world development indicators. 2019 [cited 2021 Nov 26]. https://data.worldbank.org/country/cameroon

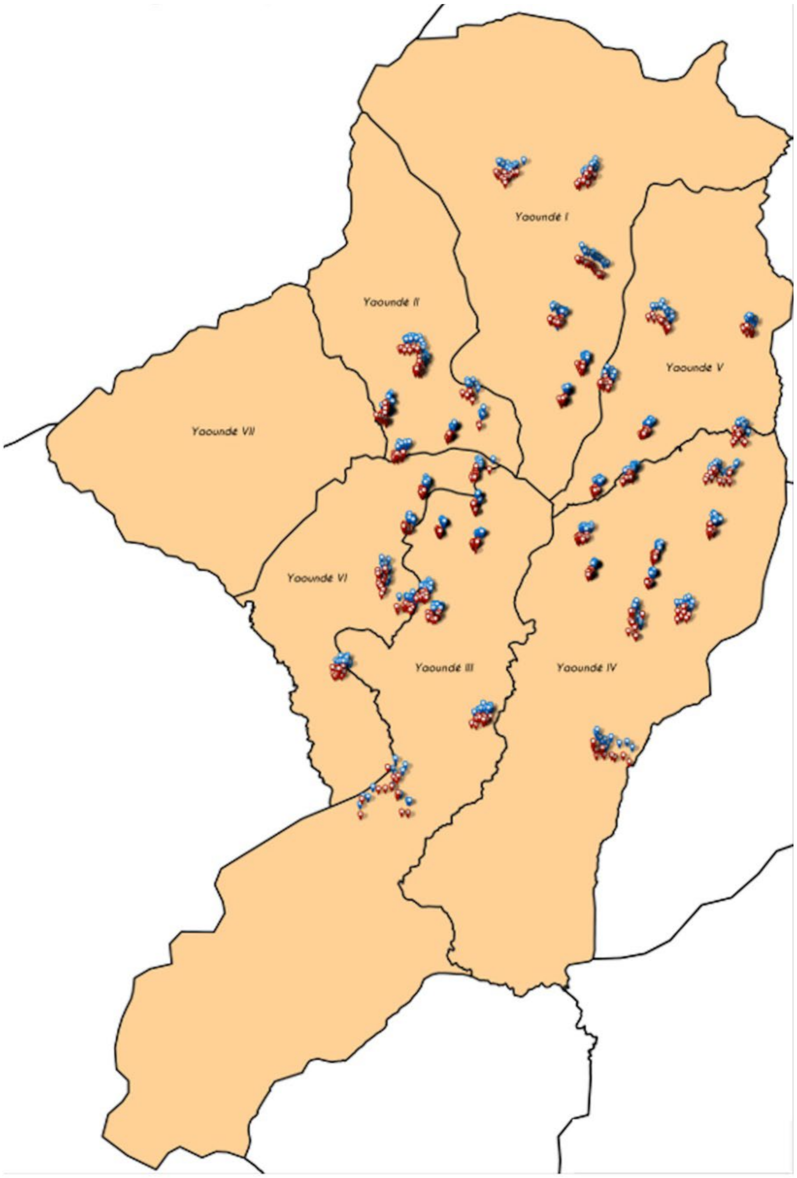

Appendix Figure 1. Map showing the locations in the different districts of Yaounde, where participants were recruited for the first (blue) and second (red) survey. Samples from the first and second survey were independently selected.

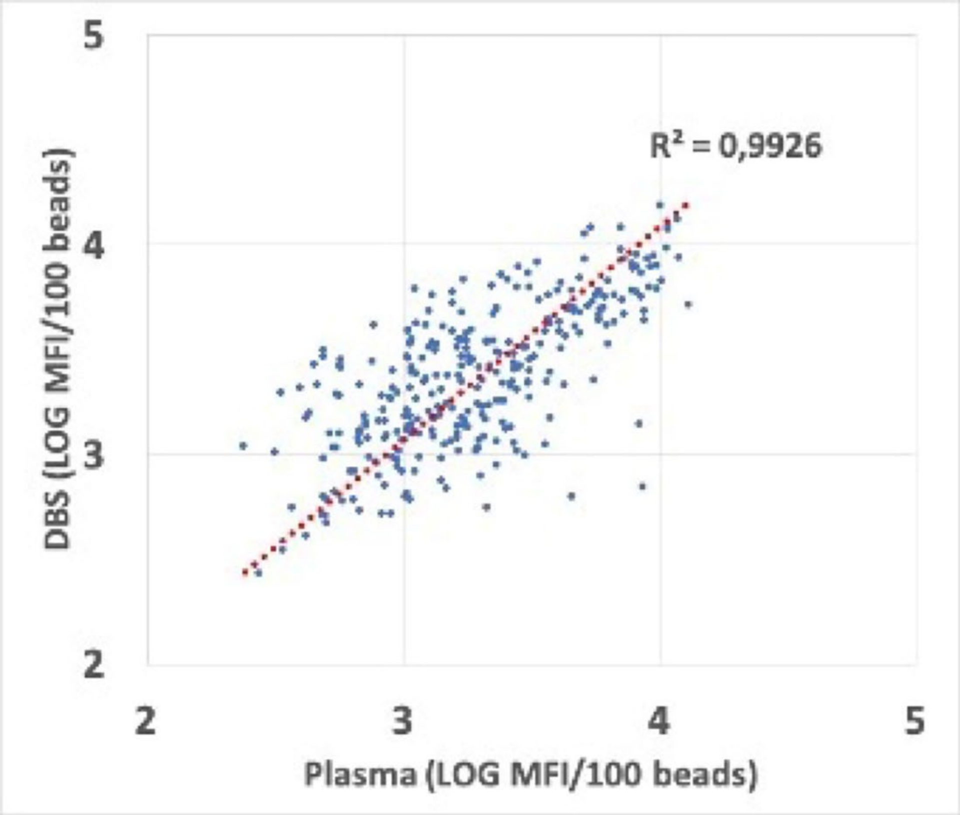

Appendix Figure 2. Correlation between MFI values from 150 paired plasma and DBS samples. Whole blood samples were collected on EDTA tubes and as DBS. Plasma was reconstituted from one DBS spot (50µl) in 1mL of incubation buffer, consisting of PBS containing 0.75 mol/L NaCl, 1%(wt/vol) BSA, 5% (vol/vol) fetal bovine serum, and 0.2% (vol/vol) Tween-20. 100 µl of diluted eluate, adjusted at a final plasma dilution of 1/200 (by taking into account hematocrit estimated at 50%), was used to test for the presence of antibodies to recombinant nucleocapsid (NC) and spike (SP) SARS-CoV-2 proteins on a Luminex platform. For each sample, Log-transformed MFI were plotted for NC and SP antigens obtained on plasma and DBS. Spearman correlation coefficient was determined and showed a high correlation between plasma and DBS on both antigens, NC and SP. Similar results were obtained on other antigens (including arboviruses and Zaire Ebolavirus, not shown). BSA, bovine serum albumin; DBS, dried blood spot; MFI, median fluorescence intensity; PBS, phosphate-buffered saline.

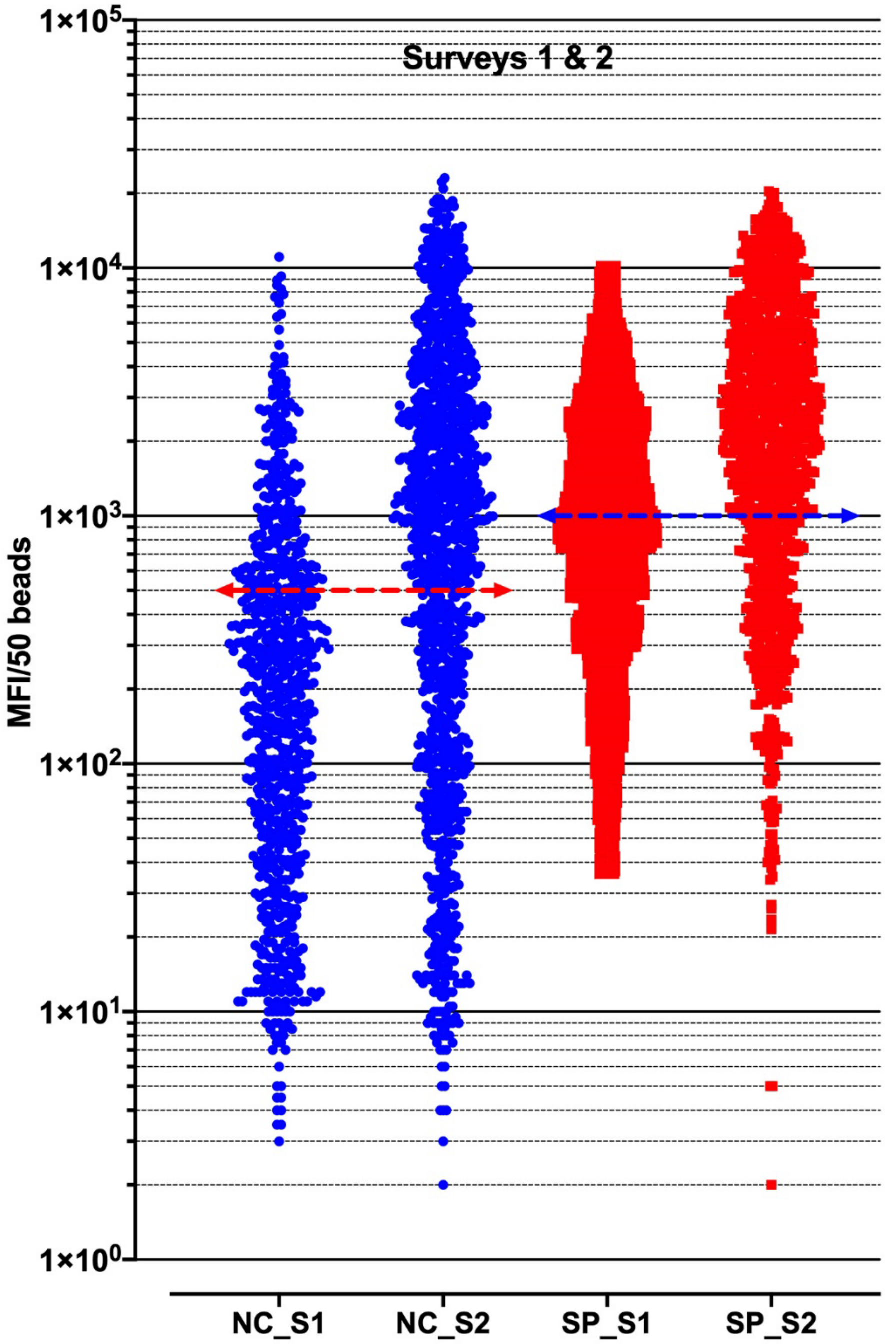

Appendix Figure 3. Distributions of MFIs for NC and SP antigens of SARS-CoV-2 for survey 1 and survey 2. MFIs for NC are highlighted in blue, the red horizontal bar corresponds to the cutoff. MFIs for SP are highlighted in red, the blue horizontal bar corresponds to the cutoff. MFI, median fluorescence intensity; NC, nucleocapsid; SP, spike.

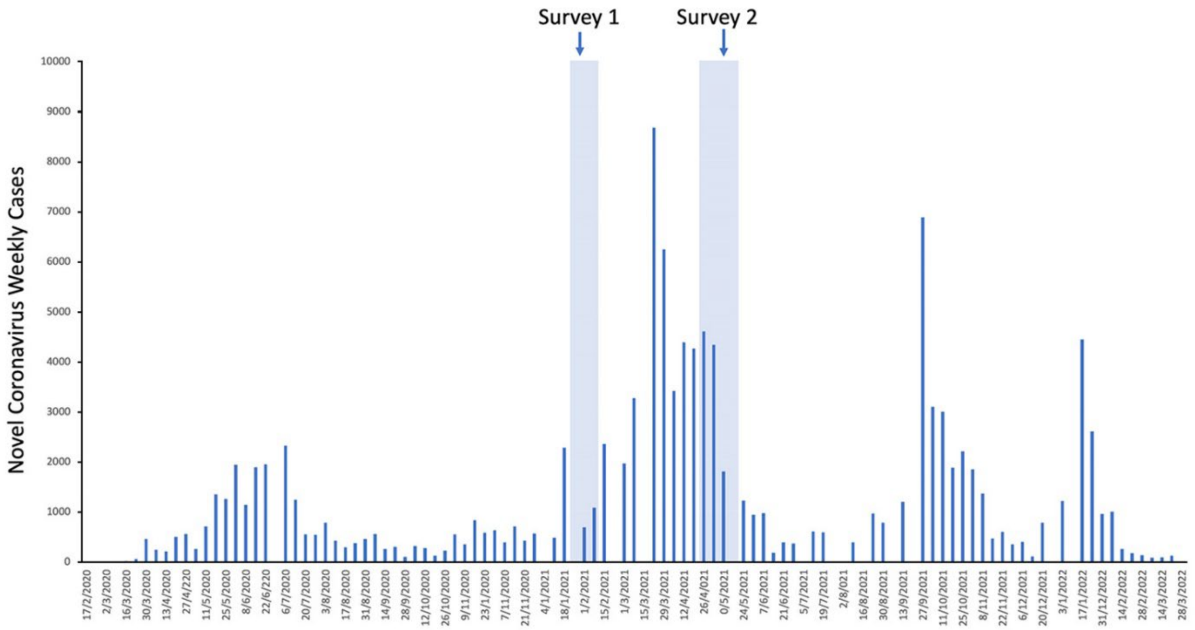

Appendix Figure 4. The graph shows the weekly reported new cases since February 2020, based on numbers reported by the World Health Organization (https://covid19.who.int/region/afro/country/cm). The transparent blue boxes and arrows indicate the time period when the 2 population-based surveys were conducted in Yaounde, Cameroon.
